# Regional and age differences in specialised palliative care for patients with pancreatic cancer

**DOI:** 10.1186/s12904-021-00870-8

**Published:** 2021-12-20

**Authors:** Mathilde Adsersen, Inna Markovna Chen, Louise Skau Rasmussen, Julia Sidenius Johansen, Mette Nissen, Mogens Groenvold, Kristoffer Marsaa

**Affiliations:** 1 Palliative Care Research Unit, Department of Geriatrics and Palliative Medicine, Copenhagen University Hospital - Bispebjerg and Frederiksberg, Bispebjerg Hospital 20D, Bispebjerg Bakke 23B, 2400 Copenhagen, NV Denmark; 2 Department of Oncology, Copenhagen University Hospital - Herlev and Gentofte, Herlev, Denmark; 3 Department of Oncology, Clinical Cancer Research Center, Aalborg University Hospital, Aalborg, Denmark; 4 Department of Medicine, Copenhagen University Hospital - Herlev and Gentofte, Herlev, Denmark; 5 Department of Clinical Medicine, Faculty of Health and Medical Sciences, University of Copenhagen, Copenhagen, Denmark; 6 Department of Public Health, University of Copenhagen, Copenhagen, Denmark; 7 REHPA, The Danish Knowledge Centre for Rehabilitation and Palliative Care, Odense University Hospital, Nyborg, Denmark; 8 Department of Clinical Research, University of Southern Denmark, Odense, Denmark

**Keywords:** Palliative care, End of life, Survival, Pancreatic cancer, Age, Geographic residence

## Abstract

**Background:**

Despite national recommendations, disparities in specialised palliative care (SPC) admittance have been reported. The aims of this study were to characterize SPC admittance in patients with pancreatic cancer in relation to region of residence and age.

**Method:**

The data sources were two nationwide databases: Danish Pancreatic Cancer Database and Danish Palliative Care Database. The study population included patients (18+ years old) diagnosed with pancreatic cancer from 2011 to 2018. We investigated admittance to SPC, and time from diagnosis to referral to SPC and first contact with SPC to death by region of residence and age.

**Results:**

In the study period (*N* = 5851) admittance to SPC increased from 44 to 63%. The time from diagnosis to referral to SPC increased in the study period and overall, the median time was 67 days: three times higher in Southern (92 days) than in North Denmark Region. The median number of days from diagnosis to referral to SPC was lower in patients ≥70 years (59 days) vs patients < 70 years (78 days), with regional differences between the age groups. Region of residence and age were associated with admittance to SPC; highest for patients in North Denmark Region vs Capital Region (OR = 2.03 (95%CI 1.67–2.48)) and for younger patients (< 60 years vs 80+ years) (OR = 2.54 (95%CI 2.05–3.15)). The median survival from admittance to SPC was 35 days: lowest in Southern (30 days) and highest in North Denmark Region (41 days). The median number of days from admittance to SPC to death was higher in patients < 70 years (40 days) vs ≥ 70 years (31 days), with a difference between age groups in the regions of 1–14 days.

**Conclusions:**

From 2011 to 2018 more patients with pancreatic cancer than previously were admitted to SPC, with marked differences between regions of residence and age groups. The persistently short period of time the patients are in SPC raises concern that early integrated palliative care is not fully integrated into the Danish healthcare system for patients with pancreatic cancer, with the risk that the referral comes so late that the patients do not receive the full benefit of the SPC.

## Background

Pancreatic cancer is one of the most lethal cancers worldwide [1]. The majority of patients with pancreatic cancer are older than 60 years and have advanced disease at time of diagnosis [2]. Despite therapeutic improvements, the prognosis of pancreatic cancer remains poor. Forty percent of the patients will die within 2 months of initial diagnosis [3, 4], and 9% are alive 5 years after diagnosis [1]. Palliative chemotherapy is the standard treatment for patients with advanced disease and has led to a benefit in overall survival [5–7]; however, the effect on quality of life (QoL) improvement is less clear. Patients with pancreatic cancer often experience complex symptomatology such as pain, fatigue, malnutrition [8] and pancreatic exocrine insufficiency, which together with treatment toxicities can contribute to a poor QoL and psychological distress [9].

Focus on integration of palliative care into oncology is increasing, and clinical trials have investigated the impact of specialised palliative care (SPC) in oncology and demonstrated improved QoL and symptom management [10–14]. Furthermore, early SPC integration into the care pathway is associated with longer survival and less aggressive oncological treatment at the end of life [15, 16].

A complex symptomatology together with a life-threating disease are the national Danish criteria for being referred to SPC [17]. However, despite national recommendations, disparities in SPC admittance have been reported [18, 19]. A nationwide study from the Danish Palliative Care Database of the 44,548 patients who died from cancer in 2010–2012 revealed that gender, age and living alone affected whether patients were admitted to SPC, regardless of diagnosis [20, 21]. Although, SPC capacity in Denmark has been reported to be associated with geographic region [22, 23], it seems relevant to investigate admittance to SPC for patients with pancreatic cancer across different regions in Denmark with the aim of increasing admittance and to optimise SPC services.

The aims of this study were to characterize SPC admittance in patients with pancreatic cancer in relation to region of residence and age.

## Materials and method

### Setting

In Denmark (5.8 million inhabitants), SPC is funded by the government and proved by palliative care teams/units at the hospitals (*N* = 25) and free-standing hospices (*N* = 19) [24]. SPC teams offer mostly outpatient palliative care services, and hospices provide mainly inpatient palliative care consultations. The provision of SPC in Denmark is about half the size recommended internationally [22, 23, 25].

### Data sources

The data sources for the present study were the Danish Pancreatic Cancer Database (started in July 2011) [26] and Danish Palliative Care Database (started 2010) [27]. Both databases use the unique civil personal registration number (CPR number) given each Danish resident, making it possible to merge the data from the two data sources.

The Danish Pancreatic Cancer Database includes all patients diagnosed with peri-pancreatic, pancreatic and duodenal cancer and is based on information obtained directly from the national registers (e.g. the Danish Civil Registration System, the Danish Pathology register and the National Patient register). It includes information on demographic factors and anticancer treatment (e.g. surgery, chemotherapy and radiotherapy) [26].

The Danish Palliative Care Database includes all patients who are referred to SPC. Health care professionals from the SPC units enter information about demographic, social and clinical factors, including patient-reported outcomes (EORTC-QLQ-C15-PAL [28]) [27].

Both databases have a very high degree of coverage and completeness [26, 27].

### Study population

The study population was based on the Danish Pancreatic Cancer Database and included 18+ year-old patients diagnosed with peri-pancreatic or pancreatic cancer in the period 1 July 2011 to 31 December 2018 who died in the same period. Patients living outside Denmark (i.e. Greenland and Faroe Island) were excluded.

In this paper, patients with pancreatic cancer are patients with peri-pancreatic and pancreatic cancer unless otherwise specified.

### Variables

The Danish Palliative Care Database and Danish Pancreatic Cancer Database contributed with the following data. Admittance to SPC (yes/no) was defined as any contact with a SPC team or a hospice either as an in- or outpatient consultation. Furthermore, the date of referral to SPC and date of first contact with SPC were obtained from the Danish Palliative Care Database.

Region of residence (Capital Region, Zealand Region, North Denmark Region, Central Denmark Region, Southern Denmark Region), sex, age, diagnosis, date of diagnosis, date of death, anticancer treatment (chemotherapy, radiotherapy, surgery) from diagnosis to death, metastatic cancer and comorbidity were obtained from the Danish Pancreatic Cancer Database.

### Statistical analyses

Using SAS statistical software (9.4) [29], we investigated the development in the proportion of patients with pancreatic cancer admitted to SPC in Denmark overall and in relation to region of residence over time. Also, time from diagnosis to referral to SPC, referral to SPC to the first contact with SPC, first contact with SPC to death and diagnosis to death were examined by region of residence and age (< 70 years vs. ≥ 70 years), reported as medians with interquartile ranges (Q3–Q1). The associations between region of residence and age and admittance to SPC were investigated using multiple logistic regression analyses including sex, oncological treatment, metastatic cancer, year of death and comorbidity. Backward stepwise selection was used. The results were reported as odds ratios (ORs) with 95% confidence intervals (CIs). The level of significance was *p* < 0.05.

### Ethics

The study was approved by The Danish Data Protection Agency (P-2020-78) and The Danish Clinical Registries (RKKP) (DPD-2020-02-04 and DPCD-2020-20-04).

## Results

### Patient characteristics

From July 2011 to December 2018, 5851 patients (18+ years old) were diagnosed with and died from pancreatic cancer in Denmark. Most patients had pancreatic cancer (DC25) (overall: 97%) and a small group of patients has peri-pancreatic cancer (DC24) (overall: 3%). Around half of the study population were men, 58% were 70+ years old, and 50% of the patients had metastatic cancer, highest in the North Denmark Region (63%), and two-thirds of the patients were registered with a comorbidity (Table 1). Fifty-eight percent were treated with one or more anticancer treatments (chemotherapy, radiotherapy and/or surgery) from diagnosis to death. Around half of the patients received chemotherapy, the lowest proportion being in North Denmark Region. Overall, three out of four patients with pancreatic cancer were referred to SPC (73%), highest in North Denmark Region (84%) and lowest in Zealand Region (65%) (Table 1).

**Table 1:** Characteristics of the patients diagnosed with pancreatic cancer who died between July 2011 and December 2018 in Denmark according to region of residence

		Total	Region of residence				
			Capital	Zealand	Southern	Central	North
		Number (%)	Number (%)				
Total		5851 (100)	1735 (30)	962 (16)	1332 (23)	1107 (19)	715 (12)
Sex	Male Female	3060 (52) 2791 (48)	854 (49) 881 (51)	521 (54) 441 (46)	688 (52) 644 (48)	616 (56) 491 (44)	381 (53) 334 (47)
Age (year)	< 60 60–69 70–79 80+	747 (13) 1664 (28) 2303 (39) 1137 (19)	214 (12) 502 (29) 662 (38) 357 (21)	141 (15) 290 (30) 384 (40) 147 (15)	154 (12) 361 (27) 542 (41) 275 (21)	154 (14) 311 (28) 448 (41) 194 (18)	84 (12) 200 (28) 267 (37) 164 (23)
Diagnosis of cancer	DC24 Peri-pancreatic DC25 Pancreatic	178 (3) 5673 (97)	21 (1) 1714 (99)	35 (4) 927 (96)	54 (4) 1278 (96)	46 (4) 1061 (96)	22 (3) 693 (97)
Comorbidity	0 1–2 3+	1980 (34) 2093 (36) 1778 (30)	499 (29) 668 (39) 568 (33)	280 (29) 324 (34) 358 (37)	505 (38) 433 (33) 394 (30)	385 (35) 436 (39) 286 (26)	311 (44) 232 (33) 172 (24)
Metastatic cancer	Yes No	2933 (50) 2918 (50)	784 (45) 951 (55)	463 (48) 499 (52)	666 (50) 666 (50)	568 (51) 539 (49)	452 (63) 263 (37)
Anticancer treatment (from diagnosis to death)	Any treatment^a^ (yes) Chemotherapy (yes) Radiotherapy (yes) Surgery (yes)	3397 (58) 3035 (52) 298 (5) 1137 (19)	1055 (61) 943 (54) 90 (5) 387 (22)	573 (60) 529 (55) 44 (5) 219 (23)	753 (57) 665 (50) 79 (6) 250 (19)	651 (59) 592 (54) 61 (6) 155 (14)	365 (51) 306 (43) 24 (3) 126 (18)
Referred to SPC^b^	Yes	4260 (73)	1251 (72)	764 (65)	862 (71)	786 (71)	599 (84)

^a^Any treatment includes chemotherapy and/or radiotherapy and/or surgery

^b^ SPC specialised palliative care

### Admittance to SPC from 2011 to 2018

In the study period, 3497 patients (60%) were admitted to SPC. The admittance for patients with pancreatic cancer increased from 44% in 2011 to 63% in 2018, highest in 2016 with 66%. In 2011, 60% of the patients with pancreatic cancer in North Denmark Region were admitted to SPC (highest level) and 32% in Southern Denmark Region. In 2018, the level had increased to 74 and 52% in these two regions. The highest increase in the proportion admitted to SPC was found in Capital Region (27%, from 39 to 66%) and the lowest increase in Zealand Region (6%, from 55 to 61%) (Table 2).

**Table 2:** The proportion of patients dying from pancreatic cancer admitted to specialised palliative care overall and by region of residence and year of death

Year	2011^a^	2012	2013	2014	2015	2016	2017	2018	Total
	% Yes (N)								
Overall admittance:	44 (89)	52 (342)	59 (453)	56 (453)	62 (495)	66 (549)	62 (556)	63 (560)	60 (3497)
Admittance in the regions of residence:									
Capital	39 (29)	45 (94)	55 (136)	55 (133)	59 (137)	65 (148)	65 (166)	66 (165)	58 (1008)
Zealand	55 (18)	61 (72)	70 (79)	64 (75)	71 (106)	72 (114)	68 (99)	61 (77)	67 (640)
Southern	32 (14)	41 (55)	53 (94)	46 (84)	54 (99)	58 (108)	52 (110)	52 (113)	51 (677)
Central	49 (16)	56 (72)	56 (75)	58 (101)	55 (80)	68 (99)	54 (89)	67 (121)	59 (653)
North	60 (12)	70 (49)	72 (69)	65 (60)	79 (73)	71 (80)	77 (92)	74 (84)	73 (519)

^a^2011 includes data from 1 July to 31 December 2011

### SPC in relation to region of residence and age

#### Time of referral

**Fig. 1:**
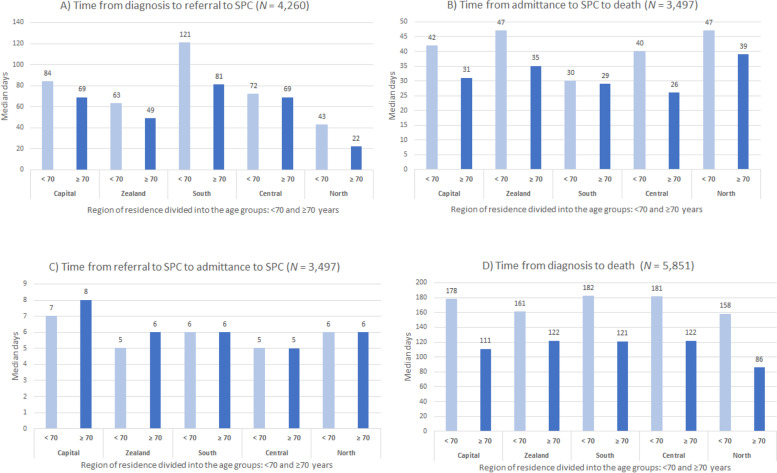
**A**) Time from diagnosis to referral to SPC (*N* = 4260), **B**) Time from referral to SPC to admittance to SPC (*N* = 3497) **C**) Time from admittance to SPC to death (*N* = 3497) **D**) Time from diagnosis to death (*N* = 5851)

For patients referred to SPC (*N* = 4260), the time from diagnosis to referral increased during the study period, except for 2017, from 23 to 97 days. Overall, patients referred to SPC were referred 2 months after diagnosis (median = 67 days) (Table 3). The time from diagnosis to referral to SPC was around three times higher in Southern Denmark Region (92 days) than in North Denmark Region (27 days). The median number of days from diagnosis to referral to SPC was lower for patients ≥70 years (59 days) vs. patients < 70 years (78 days), with regional differences between the age groups (< 70 vs ≥ 70 years) from 3 days in Central Denmark Region to 40 days in Southern Denmark Region (Fig. 1, A and Table 4).

**Table 3:** The median time in days from: diagnosis to referral to SPC, referral to SPC to admittance to SPC, admittance to SPC to death and diagnosis to death by year of death

	2011^a^	2012	2013	2014	2015	2016	2017	2018	Total
	Median (IQR^b^)								
Time from diagnosis to referral to SPC *N* = 4260	23 (37)	45 (126)	59 (215)	67 (226)	75 (261)	84 (316)	70 (266)	97 (298)	67 (235)
Time from referral to SPC to admitted to SPC *N* = 3497	4 (6)	5 (7)	5 (7)	6 (8)	6 (9)	6 (8)	7 (9)	7 (10)	6 (9)
Time from admittance to SPC to death *N* = 3497	20 (36)	35 (70)	34 (62)	31 (71)	34 (83)	39 (76)	35 (83)	39 (89)	35 (75)
Time from diagnosis to death, for all *N* = 5851	44 (57)	92 (180)	139 (238)	125 (274)	158 (298)	185 (383)	148 (347)	170 (354)	136 (278)
Time from diagnosis to death for patients referred to SPC *N* = 4260	48 (57)	111 (183)	154 (255)	156 (289)	181 (308)	203 (392)	158 (344)	208 (355)	159 (293)
Time from diagnosis to death for patients not referred to SPC *N* = 1591	40 (54)	58 (151)	107 (224)	88 (217)	81 (243)	128 (291)	122 (362)	80 (272)	82 (228)

^a^2011 includes data from 1 July to 31December 2011

^b^Interquartile Range (Q3-Q1),

**Table 4:** Admittance to specialised palliative care in relation to region of residence, age, sex, metastatic cancer, cancer treatment and year for death for patients with pancreatic cancer (*N* = 5851)

		Admittance to SPC^a^
		OR (95%CI)
Region of residence		*p = < 0.001*
	Capital	1
	Zealand	1.42 (1.20–1.69)
	Southern	0.74 (0.64–0.86)
	Central	1.04 (0.89–1.21)
	North	2.03 (1.67–2.48)
Age (year)		*p = < 0.001*
	< 60	2.54 (2.05–3.15)
	60–69	1.41 (1.19–1.66)
	70–79	1.29 (1.11–1.50)
	80+	1
Sex		*p = < 0.001*
	Male	0.71 (0.64–0.79)
	Female	1
Metastatic cancer		*p = 0.004*
	Yes No	1.18 (1.06–1.32) 1
Anticancer treatment (from diagnosis to death)		*p = < 0.001*
	Yes	1.81 (1.61–2.03)
	No	1
Year of death		*p = < 0.001*
	2011^b^	0.51 (0.37–0.70)
	2012	0.62 (0.51–0.77)
	2013	0.81 (0.66–1.00)
	2014	0.77 (0.63–0.94)
	2015	0.93 (0.76–1.14)
	2016	1.14 (0.92–1.40)
	2017	0.96 (0.78–1.17)
	2018	1

^a^Specialised palliative care

^b^2011 includes data from 1 July to 31 December 2011

#### SPC

The time waiting for SPC increased slightly from July 2011 to December 2018 (Table 3). The median number of days from referral to SPC to admittance increased from 4 to 7 days (Table 3), with minor geographic differences (5 vs 7 days). The median number of days from referral to SPC to admittance to SPC was similar in patients < 70 years vs ≥ 70 years (6 days). Minor differences between the age groups were found in the five regions (Fig. 1, B). Overall, patients with pancreatic cancer survived around 1 month (35 days) from the day of admittance to SPC, with lowest number of days observed in Southern Denmark Region (30 days) and highest in North Denmark Region (41 days). The median number of days from admittance to SPC to death was higher in patients < 70 years (40 days) vs ≥ 70 years (31 days). The difference between age groups was lowest in Southern Denmark Region (1 day) and highest in Central Denmark Region (14 days) (Fig. 1, C).

#### Time from diagnosis to death

In the study period, time from diagnosis to death for patients with pancreatic cancer (*N* = 5851) increased (median 44 to 170 days), except for 2017 (Table 3). Between the regions of residence, the median time from diagnosis to death differed from 105 days (North Denmark Region) to 142 days (in Capital Region). For patients not referred to SPC, the median survival was 82 days, highest in 2016 (128 days), decreasing to 80 days in 2018 (Table 3). The time from diagnosis to death differed between the regions: the median time from diagnosis to death for patients not referred to SPC was 32 days in North Denmark Region and 112 days in Central Denmark Region (data not shown). The time from diagnosis to death was shorter in patient ≥70 years (113 days) than in patients < 70 years (172 days). The difference in time from diagnosis to death in patients < 70 years vs patients ≥70 years differed, from 72 days in North Denmark Region (shortest time for the oldest) to 39 days in Zealand Region (Fig. 1, D).

#### Regression analysis

In the multiple logistic regression analysis the associations between region of residence and age and admittance to SPC were statistically significant. Comorbidity was not associated with admittance to SPC and it was therefore excluded from the final model. In relation to region of residence higher admittance to SPC was found in North Denmark Region (OR = 2.03 (95% CI 1.67–2.48)) and lower admittance was found in Southern Denmark Region (OR = 0.74 (95% CI 0.64–0.86)) compared to Capital Region. Further, decreasing admittance to SPC with increasing age was found; patients < 60 years had higher admittance to SPC compared to older patients (80+ years) (OR = 2.54 (95% CI 2.05–3.15)).

## Discussion

The aims of this register-based study with data from two nationwide databases were to characterize SPC admittance in patients with pancreatic cancer over time and in relation to region of residence and age. For patients with pancreatic cancer we found a clear increase in time from diagnosis to death in the study period. Nevertheless, the degree of increase in time from SPC admittance to death was not great, but marked regional differences were found, patients in Southern Denmark Region having lower admittance to SPC compared to Capital Region and the shortest time in SPC. The lowest admittance to SPC was found for older patients compared to younger. In all regions, we found that the oldest patients (≥ 70 years) had the shortest time from diagnosis to death and time in SPC, although these patients were referred earlier to SPC, i.e. closer to the diagnosis, and there was a difference between age groups that differed in relation to region.

The annual report from the Danish Palliative Care Database showed that admittance to SPC for patients with cancer is around 50% [24]. In this study, we found that 60% of patients with pancreatic cancer were admitted to SPC, which is similar to the results from previous studies that also found that patients with pancreatic cancer had higher admittance to SPC than patients with other cancer diagnoses [21, 23].

A previous Danish study investigating admittance to SPC for cancer patients referred to SPC found similar regional differences. Patients in North Denmark Region were more often admitted to SPC compared to patients in Capital Region [23]. A similar pattern was found in the present study, where patients in North Denmark Region had higher odds for admittance to SPC and further had the highest number of days in SPC.

The literature shows that younger cancer patient are more often admitted to SPC than are older cancer patients [21, 30, 31]. A study investigating cancer patients referred to SPC (and thereby patients with a need for SPC) the conclusion is similar: younger patients were more often than older patients admitted to SPC [23]. For patients with pancreatic cancer, we found that younger patients had more days in SPC compared to older patients, but older patients were referred to SPC earlier.

SPC is recommended to all patients with a complex symptomatology and life-threating disease [32]. Early integration of SPC alongside standard anticancer care is strongly preferred in patients with advanced pancreatic cancer, based on their short life expectancy and accompanying debilitating symptoms. Despite national and international recommendations, our study found that 25% had never been referred to SPC and 50% had never been admitted to SPC. Furthermore, differences of up to 20% in both SPC referral and admittance were found to be associated with geographic region, suggesting that referral criteria are non-uniform and therefore based on local considerations. At the same time, the Danish government’s national audit has also demonstrated similar inconsistencies in referral and admittance to SPC [33]. There is no common clinically useful understanding of who should be referred, when in the disease trajectory the referral is relevant, and what symptoms should lead to a specialised palliative care unit accepting a referral. As Denmark is a relatively homogeneous society, it is unlikely that these inconsistencies are solely due to differences in socio-economic patient conditions, but it is more likely that there are differences in the health professional approach to the palliative treatment of pancreatic cancer patients.

The difference in admittance to SPC and the number of days in SPC between regions found in this study should also be seen in the perspective that the provision of SPC has been found to be higher (and closer to the international standards (EAPC White Paper)) in North Denmark Region than in Southern Denmark Region [22, 23], and thereby the availability of SPC could be part of the explanation for the higher numbers of days in SPC for patients living in North Denmark Region.

Older patients were referred to SPC earlier but spent less time in SPC than younger patients. Systematisation of the referral time is an option to reduce the difference in the time in SPC. Early referral to SPC at the time of diagnosis in patients with advanced pancreatic cancer can be justified along with standard cancer care based on limited survival and the disabling nature of the disease.

Palliation is not a medical specialty in Denmark. Palliation is a health professional area of interest. This can have consequences for the development of the local professional culture and habits. Furthermore, local development strategy for palliative medicine implies implementation of standards at the national level. In addition, there is still a debate in Denmark about the extent to which palliative treatment should be performed in the basic specialties or at SPC units is units. In theory, this could also lead to some oncology departments referring either too many or too few to palliative care, thereby making it difficult to determine where the future resources for palliative care should be located.

One region had the highest percentage of patients with metastatic pancreatic cancer at time of diagnosis (63% vs. 45 to 51% in the other regions). In this region, the patients with pancreatic cancer had a shorter survival time from diagnosis to death (105 days) compared to around 140 days in the other four regions, and also a shorter time from diagnosis to referral to SPC of 27 days, whereas the other regions had 53 to 92 days. Furthermore, there was a slightly longer time in SPC of 41 days in this region, whereas it was30, 32, 34 and 40 days in the other regions.

There was a marked increase in time from diagnosis to death in the study period, which may be explained by the development of new chemotherapeutic treatment combinations [6, 7]. Nevertheless, the degree of increase in time from SPC admittance to death was not remarkable, raising concern that early palliative care was not fully integrated into the Danish healthcare system for patients with pancreatic cancer. This suggests that there is still an understanding and clinical practice of considering palliative medicine as a treatment giving to patients at the end of the disease trajectory. This is the opposite of national and international intentions regarding early implementation of palliative care.

The study is based on the national databases the Danish Palliative Care Database and the Danish Pancreatic Cancer Database, which include data of very high completeness and quality, minimising the risk of selection bias and misclassification. These databases provide a unique opportunity to investigate the disease trajectories of patients with pancreatic cancer from diagnosis to death and in relation to referral and admittance to SPC.

A limitation of the study is that the date of referral to SPC can only be entered once for each patient in relation to each SPC unit. This means that if a patient was referred to a SPC unit and rejected, but later re-referred and accepted to the same SPC unit after provision of specified information about the physical burden and/or psychological distress, then it is the last referral data that is entered into the database. This means that the time (number of days) from referral to admittance to SPC might look better than it really was and, furthermore, that the referral to SPC might appear to later in the patient trajectory than it actually was. However, the registration is identical in all geographic regions and over time, and the data are therefore useful for investigating differences between regions of residence and age groups. Furthermore, it is a limitation of the study that the need for SPC is unknown; however, we have no reason to believe that patients in one region had a higher need for SPC than patients in another region, and, therefore, we do not believe that the differences found between the regions in this study could be explained by the need for SPC.

The Danish healthcare system is almost fully tax-financed and is based on. It is made from an ideological basic idea of equal access to health care for all. The variation in referral and acceptance of referral shows a need for national standards that can ensure a uniform treatment of patients with pancreatic cancer across the country.

## Conclusion

From 2011 to 2018 more patients with pancreatic cancer were admitted to SPC, with marked differences between regions of residence and age groups. The persistent short time the patients are in SPC rises the concern that early integrated palliative care is not fully integrated in the Danish healthcare system for patients with pancreatic cancer with the risk that the referral comes so late that the patients do not receive the full benefit of the SPC.

## Data Availability

The data utilized in this study are available at Danish Clinical Registries (RKKP) [www.rkkp.dk]. Restrictions apply to the availability of these data. Contact: rkkp@rkkp.dk, Frederiksberg Hospital, Ndr. Fasanvej 57, 2000 Frederiksberg, Denmark.

## Abbreviations

SPC: Specialied palliative care
DPD: Danish Palliative Care Database
DPCD: Danish Pancreatic Cancer Database
QoL: Quality of life
